# Immunological Correlate of Protection by PHV02 Live, Attenuated Recombinant VSV Vaccine Against Nipah Virus Disease Using an Improved Neutralizing Antibody Method, with Considerations for Future Research

**DOI:** 10.21203/rs.3.rs-10860842/v1

**Published:** 2026-09-29

**Authors:** Thomas P Monath, Richard Nichols, Wasima Rida, Joan S. Fusco

**Affiliations:** Quigley BioPharma LLC; Public Health Vaccines Inc.; Public Health Vaccines Inc.; Public Health Vaccines Inc.

**Keywords:** Vaccine, recombinant vesicular stomatitis (rVSV) virus, Nipah virus, neutralizing antibody, immune correlate

## Abstract

Establishing the immune correlate of protection for a vaccine could be the basis for regulatory approval. The immune correlate of PHV02, a clinical-stage recombinant vesicular stomatitis (rVSV) vaccine against Nipah virus (NiV) disease, was investigated in the African green monkey (AGM) model. Although a mechanistic immune correlate is not required, the focus was on neutralizing antibody, which is the mediator of protection against future viral infection. Two experimental approaches were used to determine the correlation between neutralizing antibody levels, survival, and viral and clinical outcomes after 100% lethal intranasal/intratracheal (IN/IT) challenge with NiV (Bangladesh): 1) reduction in vaccine dose given 28 days before challenge; and 2) challenge at early times after vaccination. In a previous publication of the results of these studies, a neutralization test employing wild type NiV (wt NT) under high (BSL-4) biocontainment was employed. Since this is not sustainable for immunological testing in multiple clinical trials, we developed and qualified a pseudovirus microneutralization test (VNA) using a non-replicating rVSV expressing NiV F and G envelope proteins and luciferase that can be performed at BSL-2. Sera from the two studies in the AGM model were re-tested using the VNA method. There was a high correlation between the VNA and wt NT. We confirmed that reduction in PHV02 vaccine dose to very low levels led to primary vaccine failure rather than a sub-protective level of antibody, a feature common to other replication-competent live vaccines. All AGMs survived when vaccinated with PHV02 at a dose (2 × 10^7^ pfu) employed in clinical trials 21, 14 or 7 days before challenge. Neutralizing antibodies by VNA appeared by Day 13–14 after vaccination and increased by Day 20–21, reaching high titers (GMT > 1000). AGMs vaccinated 7 days before challenge had low or undetectable neutralizing antibody titers pre-challenge but also survived; these animals were on the cusp of an immune response when subjected to NiV challenge. AGMs vaccinated on Day – 21 were protected against illness whereas 4 (67%) of 6 survivor AGMs vaccinated on Day – 14 before challenge and 5 (83%) of 6 survivor AGMs vaccinated on Day – 7 before challenge had signs of illness from which they recovered. Thus, the frequency and severity of sub-lethal clinical illness decreased as time between vaccination and challenge was increased. Since high titers of VNA antibody were already present by Day 13/14, other factors such as affinity maturation of neutralizing antibody likely played a role in preventing clinical signs. A simple logistic regression model of the combined studies was used in which the sole explanatory variable was pre-challenge neutralizing antibody titers. For a pre-challenge VNA titer of 1:12 [the lower level of quantitation and representing a two-fold rise (seroresponse) from the imputed baseline of LLOQ/2], the predicted survival probability is 75–100% depending on whether random noise (± 1.0 log2) was added to the pre-challenge VNA titers. It was concluded that PHV02 vaccine elicited rapid onset of protection and that any detectable level of neutralizing antibody was a functional immune correlate of survival. A more conservative VNA titer of 1:24, representing a fourfold rise from baseline is suggested as a conservative immune correlate of protection by PHV02, thus avoiding influence of variability of the assay. By 14–21 days after vaccination VNA GMTs are many fold higher than the 1:24 threshold for protection. Various suggestions for future research aimed at confirming the PHV02 immune correlate are discussed.

## 1. Introduction

Demonstration in randomized, controlled clinical trials that a high proportion of participants mount an immune response reaching the threshold for protection could be the basis for marketing authorization of a new vaccine. This concept is embodied in the US Food & Drug Administration (FDA) “Accelerated Approval” pathway based on an immunological biomarker that predicts clinical benefit.^1,2,3^

Many vaccines have been approved based on an immune surrogate including those for hepatitis B, COVID-19, influenza, pneumococcal and meningococcal disease, smallpox, rabies, yellow fever, and Japanese encephalitis, but in many cases, it has been possible to compare the immune responses to those elicited by a previously approved vaccine with established clinical effectiveness, using a non-inferiority design.

In some cases, it may be possible to infer the protective threshold of protection from immune responses to the natural infection, as exemplified by SARS-CoV-2^2^ and from instances of vaccine failures informed by an inadequate antibody response.^3^ However, for a new vaccine against a rare disease for which there are no quantitative data on the relationship of recovery and survival to the immune response in human patients and for which there is no approved vaccine for a non-inferiority study, evidence from well-established animal models plays a key role in regulatory decisions.^4^ Animal models may be used to establish protective antibody threshold by means of active immunization^5,6^ or by passive transfer of immune serum.^7,8^

The neutralization test is a functional (mechanistic) assay, the gold standard for estimating protection against most viruses, and importantly, is more sensitive than IgG ELISA^9,10,11,12,13^ with sensitivity being the critical parameter if a threshold of protection at a low level of antibody is sought. A mechanistic correlate of protection is not mandatory for regulatory approval but is preferred.^14^

Nipah virus (NiV) disease is a rare, but highly lethal bat-borne zoonosis occurring in sporadic cases and repeated outbreaks in south and southeast Asia^15^ caused by an RNA virus in the Henipavirus genus, family *Paramyxoviridae*. Development of NiV vaccines is a high priority for the World Health Organization (WHO)^16^ and the Coalition for Epidemic Preparedness Innovations (CEPI).^17^ Multiple NiV vaccines are in development, and a number have entered Phase 1 or 2 clinical trials [a subunit protein vaccine (NCT04199169), an mRNA vaccine (NCT05398796), a chimpanzee adenovirus vectored vaccine and a recombinant vesicular stomatitis (rVSV) vectored live, attenuated vaccine (NCT05178901)], which is the vaccine candidate described here.

PHV02 is a live, attenuated rVSV in which the glycoprotein (G) gene of VSV (the principal neurovirulence factor) has been deleted and replaced with the corresponding envelope glycoprotein genes of both Ebola virus (Kikwit) (EBOV GP) and Nipah virus (Bangladesh) (NiV G). The EBOV GP is required for fusion and cell entry, which are not mediated by the NiV G protein. The NiV G protein is responsible for attachment to cell receptors, principally ephrin B2 and B3 and heparan sulfate^18^, and antibodies against G protein neutralize both the recombinant vaccine virus and wild-type NiV by preventing attachment to cells.^19^ Despite the simplicity of this concept, how antibody binding results in virus inactivation is incompletely understood despite decades of research.

Use of rVSV-EBOV as the vector for immunization against NiV has the important advantage of providing an extensive backdrop of data concerning safety and efficacy (including the threshold for protection) of the Ebola vaccine (Ervebo^®^), against which the NiV vaccine can be compared.

The grivet monkey (*Chlorocebus aethiops)*, often referred to as an African green monkey (AGM) is an established model of NiV disease in humans.^20^ A number of studies in AGMs have demonstrated that the PHV02 rVSV vaccine elicits neutralizing antibodies and protects against 100% lethal NiV challenge by the mucosal route (mimicking natural infection).^21,22,23,24^

In a previous publication, we described studies of PHV02 vaccination of AGMs. We dissected the relationship between neutralizing antibody levels and protective immunity.^25^ Two studies with different designs were performed. In the first (“down-dosing”) study, AGMs were vaccinated with descending doses of PHV02, underwent weekly testing for antibody responses and were then challenged with a lethal dose of NiV at 28 days after vaccination. In the second “(time to protection”) study, AGMs were vaccinated with PHV02 and challenged 21, 14 or 7 days after vaccination.

In both studies, antibody responses and their correlation to clinico-pathological outcomes were initially measured by a neutralization test using wild-type NiV (wt NT),^37^ a process requiring BSL-4 containment. The biocontainment requirement made bridging to clinical trial results extremely difficult. Moreover, the wt NT employing a constant virus-serum dilution method for reduction in cytopathic effects (CPE) was found to be relatively insensitive, probably because a high concentration of antibody is required to block the highly cytopathic wt virus. When a luciferase-based Virus Neutralization Assay (VNA) was substituted using a non-replicating rVSV pseudovirus expressing NiV F and G proteins and luciferase and clinical trial samples were re-tested, the VNA was found more sensitive than the CPE-based wt NT method.^26^ The VNA method can be performed with standard BSL-2 procedures and is now routinely used for immunogenicity testing of PHV02 in nonclinical studies and in clinical trials.

To reinvestigate the studies in the AGM model by using the VNA, we re-tested all samples from the previous studies^33^ and correlated results to the clinico-pathological outcomes. The data provide further visibility on the immune correlates of protection in the nonhuman primate model and enable bridging these data to those from previous and future clinical trials that employ the VNA. In this paper, we describe the VNA method, reanalyze, and reinterpret results of the AGM studies using the assay. So informed, we discuss how immune correlates of protection might be investigated and confirmed in future studies.

## 2. Materials and Methods

### Vaccines and challenge virus

PHV02 (rVSV?G-EBOV GP-NiV G) was manufactured for PHV by Ology Bioservices Inc., Alachua FL, which was subsequently acquired by National Resilience LLC. The vaccine used in Study 1 was Lot PTR3.230 prepared for use in toxicology studies. The PHV02 vaccine used in Study 2 was GMP Clinical Trial Material, Lot No. 46982.

The control vaccine rVSV?G-EBOV GP [rVSV-EBOV (Kikwit 1995)], similar to the approved Ebola vaccine (Ervebo), was provided by the Rocky Mountain Laboratories, National Institutes of Health. Both PHV02 and the control viruses are replication-competent and highly attenuated in various animal models compared to the parental viruses (i.e. VSV, EBOV, NiV).^27^ Both vaccine viruses are produced in Vero cell cultures. The NiV challenge virus is a human isolate, Nipah (Bangladesh/200401066, GenBank AY988601) passaged 3 times in Vero cells and frozen at ≤−70°C in Dulbecco’s Minimum Essential Medium-10% heat-inactivated fetal bovine serum.

### Animal procedures.

Studies in AGMs were conducted at the Rocky Mountain Laboratories, National Institutes of Health (NIH). All animal procedures followed NIH’s *Guide for the Care and Use of Laboratory Animals* and USDA’s *Animal Welfare Act*. All animal procedures were reviewed and approved by the institution’s Animal Care and Use Committee (ACUC). AGMs were procured from a commercial vendor. All experimental manipulations were performed on anesthetized animals. Animals were anesthetized with IM Ketamine/HCl (8–20 mg/kg) or Telazol (2–6mg/kg, IM). Diazepam (0.25–1mg/kg) IM or Midazolam (0.05–0.15 mg/kg IM) was used, as required, for additional sedation along with Ketamine or Telazol. Inhalational Isoflurane (1–5% vaporizer setting) was used for maintenance of anesthesia during prolonged experimental examinations. Isoflurane was administered via a mask using a calibrated vaporizer. All nonpharmaceutical grade materials selected for use were in accordance with *Guidelines for the Use of Non-Pharmaceutical Grade Compounds in Laboratory Animals*. Animals were euthanized by exsanguination via intracardiac needle and/or transection of the femoral artery following induction of deep anesthesia or by intravenous or intracardiac administration of 1ml/5kg euthanasia solution (pentobarbital/phenytoin) while under anesthesia.

AGMs were randomly divided into treatment groups and allowed to acclimate to the study room for a ≥ 5 days before going on study. Animals were vaccinated IM in the caudal thigh and held for varying intervals before NiV challenge. Monkeys were monitored at least twice daily for adverse effects after vaccination and signs of disease after challenge. At protocol-specified intervals, thoracic radiographs, blood, and swabs (nasal, oral, and in some animals, rectal) were collected for clinical laboratory, virology and immunological tests. These methods are described in more detail in the original publication^25^ and are not reiterated here.

### Nipah virus challenge.

Under anesthesia, AGMs were challenged with 2 × 10^5^ 50% tissue culture infectious doses (TCID50) NiV (Bangladesh) divided equally between intratracheal (IT) inoculation via an endotracheal tube and intranasal (IN) inoculation by slowly dripping the 0.5 mL of inoculum into each nare with a micropipette.

### Clinical endpoints and euthanasia.

Experienced staff observed AGMs and recorded clinical signs using a semiquantitative scoring sheet. Any AGM with a clinical score ≥ 35 was euthanized.

### Thoracic radiographs.

Ventro-dorsal and lateral thoracic radiographs were done under anesthesia and were scored for pulmonary infiltrates by two boardcertified laboratory animal veterinarians according to a standard scoring system.^28^ Each lung lobe was scored individually based on the following criteria: 0 = normal examination; 1 = mild interstitial pulmonary infiltrates; 2 = moderate interstitial pulmonary infiltrates, perhaps with partial cardiac border effacement and small areas of pulmonary consolidation (alveolar patterns and air bronchograms); and 3 = pulmonary consolidation. The scores assigned to each of the six lung lobes were summed and recorded as the radiograph score on a given day. Scores may range from 0 to 18 for each animal on each examination day.

### Neutralization.

In the previously published study^25^, the neutralization assay was a constant virus-serum dilution method employing wild-type NiV performed under BSL-4 containment at RML. Serial twofold dilutions of heat-inactivated serum in Dulbecco’s minimal essential media with 2% heat inactivated fetal bovine serum, 1 mM L-glutamine, 50 U/ml penicillin and 50 μg/mL streptomycin were mixed with 100 TCID_50_ of NiV (Bangladesh) virus, incubated (37° C, 60 min) and inoculated onto monolayer cultures of Vero E6 cells in 96 well plates. The starting serum dilution after adding virus was 1:5. The neutralization titer is the highest dilution of serum for which no CPE are observed.

The neutralization method was changed to a nonpathogenic pseudovirus assay (VNA) performed at BSL 2 by Q2 Solutions (now IQVIA), Durham NC. The pseudovirus used in the VNA is a recombinant VSV with the VSV G glycoprotein removed and replaced with the Nipah (Bangladesh) and fusion (F) proteins as well as a reporter gene (luciferase). Serum is diluted in serial twofold steps from 1/6 to 1/384 and mixed with an equal volume of diluted pseudovirus for final serum dilution of 1/12 to 1/768. Neutralization is allowed to proceed for 60 ± 5 min at 37°C after which the serum-virus mixture is inoculated onto Vero cells (ATCC CCL-81) grown in 96 well plates. Inoculated plates are incubated at 37 ± 2°C with humidity and 5 ± 3% CO_2_ for 20 ± 2 hours. Luminescence is detected by using the ONE-Glo^™^ luciferase assay system using the Cytation- 5 plate reader and the neutralization titer reported as the highest reciprocal dilution of serum at which 80% of the luciferase signal is inhibited. The lower limit of quantitation (LLOQ) is a titer of 12. For a negative pre-vaccination sample, a titer of LLOQ/2 (6) is imputed.

### IgG ELISA.

IgG ELISA was performed by RML as described previously.^25^ Since this report concerns re-examination of the neutralizing antibody responses by a different method (VNA) and correlations with survival and clinical outcomes, we do not reiterate results by ELISA, which can be found in the original publication. The RML IgG ELISA was less sensitive than wt NT or VNA for detection of antibody. IgG ELISA was also performed on AGM samples at Q2 Solutions (now IQVIA Laboratories) using a different procedure and, although correlated to VNA was also less sensitive than it (data not shown).

### Quantitative PCR.

RNA was extracted from swab samples and tissues and tested by real-time RT-PCR targeting the NiV Bangladesh N gene of NiV as described in the original publication.^33^ Only results relevant to the current paper are reported.

### Statistical methods.

Immunogenicity data were summarized by geometric mean titer (GMT) and geometric mean standard deviation (GSD) for each study by treatment group and pre-challenge time point. For titers below the lower limit of quantitation (LLOQ), LLOQ/2 was imputed before calculating summary statistics. Seroconversion was defined as a 4-fold or greater increase in titers from baseline defined as the last time point prior to vaccination. Linear regression analyses of log2-transformed VNA titers on log2-transformed wt neutralizing titers were performed by study and time point post-vaccination to assess the concordance between assays. Spearman’s rank correlation coefficient is reported for each analysis. Logistic regression analysis of survival on pre-challenge log2-transformed VNA titers was performed in which data from both studies were pooled including control monkeys. Confidence intervals are based on the nonparametric bootstrap method. Two thousand bootstraps were performed in which the 2.5th and 97.5th percentile constitute the 95% CI for a given pre-challenge VNA titer. This approach gives asymptotically correct coverage even if the parametric model does not fit the data^29^. Due to tied data arising from multiple monkeys having pre-challenge titers below the LLOQ, a sensitivity analysis was performed in which a small amount of random noise was added to the log2-transformed VNA titers. The random noise had a uniform distribution from – 0.5 log2 to 0.5 log2.

For Study 1, a one-way analysis of variance was performed to assess the effect of dose on pre-challenge VNA titers. All p-values are two-sided. Analyses were performed with the open-source program R version 4.6.0 (https://www.Rproject.org/) or with GraphPad Prism 10.

## 3. Results

### Virus Neutralization Assay (VNA)

The VNA assay was fully qualified. Repeatability (intra-assay) had a 27.2% coefficient of variability (CV) and intermediate precision (assays on different days with different plates and different analysts) had a 33.6% CV. The Quality Control range was determined. One positive control and one negative control were tested during assay qualification in 12 different runs and geometric mean values and corresponding ± 2SD were calculated. The initial qualification was repeated to obtain a 5% failure rate on the CV of the virus control and an internal quality control (IQC). The lower limit of quantification (LLOQ) of the VNA was determined from the precision and linearity assessments during the assay qualification and is a value of 12. For calculation of seroresponse (≥ 2-fold increase) and seroconversion (≥ 4-fold increase) rates, geometric mean titer (GMT) and geometric mean fold increase (GMFI), LLOQ/2 is used as the baseline, a value of 6.

Serum samples from the two experiments in AGMs performed at RML^25^ were transferred to IQVIA Laboratories and retested using the VNA. Only sera from experimental animals obtained before NiV challenge outside the BSL-4 area at RML were transferred for testing. Therefore, post-challenge sera were not tested.

To define the relationship between VNA and the wt NT used in the original studies, we performed a linear regression analysis of AGM sera (Fig. 1). There was a high correlation between the two neutralization assays. The VNA titers were about 5 times higher than wtNT.

### Nipah virus challenge in control AGMs

All AGMs that received the control rVSV-EBOV vaccine (lacking the gene for NiV G protein) and were challenged with NiV (Bangladesh) developed viremia (3–6 log10 RNA copies/mL), clinical signs of illness and hematological and biochemical test abnormalities, lung radiograph changes, and died or met euthanasia criteria by day 7 or 8.

NiV challenge in control AGMs was uniformly fatal and accompanied by physiological, clinical, virological and histopathological changes indicating fulminant NiV infection. These outcomes are consistent with previous descriptions of NiV in the AGM model.^21,30,31^

The quantity of NiV used for IT/IN challenge in our studies is 28–100-fold higher than was shown to cause lethal infection in AGMs challenged IT in previous studies.^32^ While the NiV dose required to infect humans is unknown, the outcome in humans (40–75% case fatality rate) is lower than in the AGM model (100%) and survival time is longer in humans.^33^ Use of a uniformly lethal, severe challenge model provides assurance that the immune correlate of protection does not represent an underestimate for humans.

### Study 1 (down-dosing): Survival in vaccinated AGMs is associated with an ‘all-or-nothing’ immune response

In the first (down-dosing) study, groups of 4 adult (> 3 kg) male and female AGMs were given a single IM inoculation of graded doses of PHV02 (High dose: 1.7 × 10^6^; Mid-dose: 1.8 × 10^4^; or Low-dose: <6.6 × 10^2^ pfu based on back-titration. The Low dose was below the LLOQ of the assay and is therefore designated as 3.3 × 10^2^ pfu/mL, since no virus was observed on back titration of the material used for vaccination. Four control AGMs were inoculated IM with rVSV-EBOV vaccine (2 × 10^7^ TCID_50_/ml). AGMs were challenged with NiV on Day 28 post-vaccination.

100% of the AGMs in the High and Mid PHV02 dose groups survived and had no clinical signs or detectable viremia after challenge, whereas 2 (50%) of 4 animals in the Low dose group and all 4 rVSV-EBOV controls died or were euthanized by Day 7 (Table 1.). Survival in the High and Mid dose groups was statistically higher than in the rVSV-EBOV controls (p = 0.0047, log rank test), but there was no difference between the PHV02 Low dose and control group (p = 0.1573). All surviving animals were also protected against clinical signs of illness and had low clinical and thoracic radiograph scores (Table 1).

Time to death/sacrifice (Day 7) in the two non-survivors in the PHV02 Low dose group was unaffected by vaccination indicating that the down-dosing design, at least in this study, did not provide a means to regulate survival rate, an effect not unexpected for a replicating virus where a low vaccine dose can either initiate an infection and immune response or fail to do so. When a very low (and indeterminate vaccine dose below the level of quantitation) was given, there were 50% survivors, suggesting an all-or-nothing outcome. This topic will be discussed in more detail below.

All survivor AGMs mounted a substantial neutralizing antibody response measured by VNA before challenge (Table 1).

The VNA response to vaccination was rapid and produced high GMTs (Fig. 2). There were no differences in antibody titer between the dose groups. Among survivors, the GMFI from baseline in the survivors was also similar across the three dose groups (126, 171, 176, respectively). The VNA GMT increased only modestly between Day 14 and Day 28, with GMFIs of 2.1, 3.7, and 2.1, respectively.

AGMs with any detectable concentration of VNA antibodies before challenge not only survived but were also protected against viremia (Table 1), clinical illness (Fig. 3) and radiographic evidence of pneumonia (Table 1).

The two AGMs in the Low-dose (3.3 × 10^2^ pfu) group that succumbed to infection failed to develop detectable VNA antibodies before challenge and subsequently developed high viremias, clinical and radiographic scores. These animals are considered primary vaccine failures.

It was concluded that the effect of lowering the vaccine dose was principally on antibody response rate rather than antibody kinetics, titer or fold rise; i.e., at very low dose levels, animals either responded or failed to respond and among those that responded, dose level had no effect on antibody titer or kinetics of the response. Thus, down-dosing led to primary vaccine failure in 2 of 4 animals in the lowest dose group but did not elicit a sub-protective level of antibody.

### Study 2: Time to Protection- challenge during the early immune response defines the limits of protective immunity

In Study 2, vaccination was performed 7, 14 or 21 days before challenge, with the objective of determining correlates of protection during the early phase of the adaptive immune response when antibody titers were rising. Given the dichotomous all-or-nothing pattern of survival and immune response observed in down-dosing Study 1, the time-to protection experimental design was considered more likely to reveal a threshold of protective antibodies.

Groups of 6 AGMs were vaccinated IM with 2 × 10^7^ pfu of PHV02 (a dose used in Phase 1 clinical trials) 21, 14 or 7 days before lethal IN/IT NiV (Bangladesh) challenge (Day 0). Four control AGMs received 2 × 10^7^ pfu rVSV-EBOV vaccine 7 days before challenge.

After vaccination with PHV02, neutralizing antibody was measured at different times by VNA. As for Study 1, the lower limit of quantification (LLOQ) in the VNA was determined from the precision and linearity assessments during the assay qualification and is a value of 12 (for analysis of GMT and GMFI, LLOQ/2 is used, a value of 6).

Results are summarized in Table 2.

#### Survival.

In Study 2, all AGMs vaccinated with 2 × 10^7^ pfu of PHV02 21, 14 or 7 days before challenge survived, whereas the rVSV-EBOV controls vaccinated with 2 × 10^7^ pfu 7 days before challenge succumbed or were euthanized on Day 8 after challenge.

AGMs vaccinated 21 days before challenge developed high titers of VNA neutralizing antibodies (GMT 1168.8) and were solidly protected, having no post-challenge viremia, minimal radiographic evidence of pneumonia, and minimal clinical signs (Fig. 4); 4 (67%) of the 6 animals also had no rise in neutralizing antibodies by wt NT after challenge (Table 2).

AGMs vaccinated on Day – 14, also survived and had also mounted high titers of neutralizing antibodies (GMT 302) before challenge, albeit ~ 4-fold lower than those vaccinated on Day – 21. These monkeys also had no detectable viremia after challenge but developed clinical and radiographic evidence of NiV infection (Fig. 4) which they were able to overcome. The clinical and radiographic scores in the vaccinated animals were lower than in the controls. Only 1 of 6 animals failed to develop a wt NT antibody rise after challenge (these samples could not be removed from BSL-4 containment for VNA testing).^25^ The rise in antibodies after challenge was likely due to both the evolving antibody response to the original vaccination and to the challenge infection.

AGMs vaccinated on Day – 21 and Day – 14 with respect to NiV challenge developed neutralizing antibodies by VNA between 7 and 14 days after vaccination (Fig. 5, Table 2). VNA GMTs [geometric mean standard deviation (GSD)] on Day 13–14 after vaccination were between 302.0 (3.62) and 592.6 (2.40). The neutralizing antibody response occurred principally between 7–14 days after vaccination, with a GMFI (GSD) on Day 14 of 50.3 (2.62)– 90.7 (2.49) from baseline. Thus, the antibody response was rapid and well-established by Day 14 as also seen in Study 1 (Table 1). A rapid antibody response after a single dose is typical of live viral vaccines.

The group of most interest immunologically are the AGMs that survived when vaccinated only 7 days before lethal challenge. None of these 6 monkeys had neutralizing antibodies detectable by VNA at the time of challenge. Even though the VNA appears more sensitive than wt NT, 2 (33%) of the 6 AGMs vaccinated 7 days before challenge had low but detectable antibody pre-challenge by use of wt NT performed at RML in which the lowest dilution of serum after adding virus was 1:5.^25^ It is likely that animals with no detectable antibody would be found positive if sera were tested in the VNA assay at a lower starting dilution than 1:12. This point is elaborated further in the Discussion. No protection was afforded by non-specific (innate) immunity, since all controls given the live, replicating rVSV-EBOV vector on Day – 7 died (Day + 8).

Despite having minimal or undetectable neutralizing antibody response by the time of challenge, only 1 of 6 animals vaccinated on Day – 7 developed a low viremia after challenge. All had clinical signs of infection (Fig. 4) as well as radiographic changes (Table 2), which they were able to clear. All 6 monkeys vaccinated on Day – 7 had a rise in antibodies after challenge. As pointed out in the original publication^25^ the post-challenge memory response (measured by wt NT) may have contributed to survival of the AGMs vaccinated on Day – 7. Due to biocontainment issues the post-challenge samples could not be tested at BSL-2 by VNA.

### Relationship of antibody level to survival and clinical signs.

Figure 6 shows the predicted survival curves and their 95% confidence interval for a given pre-challenge log2-transformed VNA titer for the pooled data consisting of all 38 monkeys.

For the first regression analysis, the predicted survival probability is 100% for a pre-challenge titer of 12 with 95% CI of (95%, 100%). However, when a small amount of random noise is added to the log2-transformed titers, the probability of survival becomes 61% with 95% CI (40%, 95%). The predicted survival probability increases to 81% for a pre-challenge titer of 24 with 95% CI (66%, 100%). Of note, for a pre-challenge titer of 6, the probability of survival is 38% with 95% CI (10%, 65%) suggesting a modest level of protection in monkeys with undetectable VNA titers.

Overall, the data indicates that any detectable neutralizing response before exposure to NiV is predictive of survival. Furthermore, the absence of a response when vaccination occurs shortly before exposure does not predict lack of survival, since 4 of 6 monkeys challenged

### 7 days after vaccination had no detectable neutralizing antibodies by VNA but survived.

#### Clinical scores.

AGMs vaccinated 7 or 14 days before challenge had sublethal infections of clinical significance from which they recovered (Fig. 4). All rVSV-EBOV controls had high clinical scores after NiV challenge (Fig. 4). AGMs vaccinated on Day – 21 were protected against illness whereas 4 (67%) of 6 survivor AGMs vaccinated on Day – 14 before challenge and 5 (83%) of 6 survivor AGMs vaccinated on Day – 7 before challenge had signs of illness. Thus, the frequency and severity of sub-lethal clinical illness was reduced as the time between vaccination and challenge increased. However, the magnitude of clinical illness, reflected in the numerical scores was less in vaccinated than control animals.

We had previously performed a linear regression analysis of the maximum clinical scores in Studies 1 and 2 as they relate to pre-challenge neutralizing antibody titer by wt NT and reported a moderate inverse correlation.^25^ This analysis was repeated using VNA titers, and a moderate correlation was confirmed (Fig. 3).

#### Thoracic radiographic scores.

In Study 1, survivors had lower pulmonary infiltrates caused by NiV challenge infection than controls (Table 1). In Study 2, 2 (50%) of 4 AGMs in the control group did not develop pulmonary infiltrates, making the association with antibody response and survival less clear.

#### Tissue RNA levels at necropsy.

Necropsies were performed on vaccinated survivors on Day + 41 or + 42 after challenge on Day 0 and on Low dose PHV02 non-survivors and rVSV-EBOV controls at time of death/euthanasia (7 or 8 days after challenge). In Study 1, all controls and the two non-survivors in the PHV02 Low dose group had high levels of RNA (5–10 log_10_ copies/g) and infectious virus (3–6 log_10_ TCID_50_) in multiple tissues including lung, spleen, and brain on necropsy on Day + 7.

In Study 2, all rVSV-EBOV controls had high levels of genomic RNA (4.8–9.5 log_10_ copies/g) in multiple tissues on necropsy on Day 8 after challenge, especially in lung segments, as well as brain, spleen and urinary bladder. In vaccinated AGMs, substantially lower levels of detectable genomic RNA (3–4 log_10_ copies/g) were present in one or more tissue samples, with no differences across the Day – 21, −14, and – 7 vaccine groups.

## 4. Discussion

Our studies indicate the feasibility of identifying an immune surrogate of protection for PHV02 vaccine, although considerable further effort will be necessary to meet regulatory requirements for approval. As is the case for essentially all vaccines^6^, antibodies are the principal mediator of protection against NiV.^34,35^ This has been corroborated in animal models by passive transfer of serum polyclonal antibodies^36^ and by human monoclonal antibodies with neutralizing activity^37^, which have been characterized at the epitope level.^38,39,40^. In the case of active immunization with a subunit vaccine, studies in the AGM model have estimated a protective threshold titer of 1:80 for pseudovirus neutralizing antibody.^41^ Our studies indicate a lower threshold of a titer of 1:12–24 in the VNA, consistent with estimates for the immune correlate of protection for rVSV-EBOV.^8,43^ However, live viral vaccines like PHV02 are more likely than subunit vaccines to elicit a strong non-specific (innate immune) and specific CD8 + T cell responses; it is reasonable to assume that the lower antibody level associated with protection by PHV02 than the subunit vaccine may reflect that cellular mechanisms in addition to humoral antibody are stimulated.

We previously published studies aimed at defining the Nipah virus neutralizing antibody threshold for protection by PHV02 vaccine in the established AGM model using the wt NT method.^25^ Subsequently, as progress was made in developing a VNA method to support clinical trials, it was considered important re-evaluate the results of these nonclinical studies. Therefore, the sera from the published studies were retested by VNA. We showed that the VNA assay is fit-for-purpose, and it has been used in support of Phase 1 clinical trials. Formal validation is planned to support further animal studies and a Phase 2 clinical study.

Although attenuated vaccine viruses and pseudoviruses are commonly employed in neutralization assays used in clinical research and in trials for regulatory approval, there is always a hypothetical question of whether their use biases interpretation since the virus in the test is homologous with or closer antigenically to the vaccine than the agent causing disease. It was therefore useful to show that results with the VNA correlated well with the wt NT (Fig. 1). Unsurprisingly, the interpretation of results of the AGM studies with the VNA and wt NT methods were consistent.

The parental rVSV-EBOV vaccine lacking NiV G protein expression was used as a control vaccine. As expected, none of the rVSV-EBOV control animals had a detectable NiV-specific specific immune response by VNA assay at the time of death or euthanasia. Although we did not include controls that received a true vaccine placebo, the time to death or euthanasia (7–8 days) in rVSV-EBOV controls is consistent with previous studies in the AGM NiV model^42 ,43^ suggesting that the innate immune responses to rVSV-EBOV did not significantly modulate the outcome of NiV challenge.

Compared to humans, NiV causes more active infection which is 100% lethal in the AGM model than in humans (40–70% lethal). Not unexpectedly therefore, antibody GMTs 28 days after IM inoculation with a dose used in clinical trials of PHV02 (2 × 10^7^ pfu) were approximately tenfold higher in AGMs^25^ than observed in humans (PHV, unpublished). This discrepancy in immunogenicity raises the question of the validity of conclusions from the monkey model to the immune correlate for humans. However, the results from our studies showed that a VNA titer below the limit of detection in AGMs on Day 7 was protective. The initial dilution of test serum in the VNA assays prevented detection of neutralizing antibody below a titer of 1:12. Survival in AGMs that were challenged on Day 7 on the cusp of a detectable immune response is consistent with analysis rVSV-EBOV in the AGM model of rVSV-EBOV and in humans in which a two-fold or greater increase in antibody from before to after vaccination (i.e. a 2-fold seroresponse), is associated with protection.^8,44^ Since protection is observed at minimal antibody titers, concerns are reduced about a difference in immunogenicity of the vaccine in nonhuman primates vs. humans invalidating the estimation of a protective threshold.

Linear regression analysis of the historical wt NT titers vs. VNA titers for all animals and timepoints revealed that the VNA assay was approximately 5-fold higher than wt NT titers (Fig. 1). However, that increased sensitivity did not translate into improved ability to detect very low titers as seen in animals 7 days after vaccination, possibly due to the starting final dilution of serum at 1:12.

It is not surprising that a very low level of neutralizing antibody can be protective. In field studies of mumps vaccine, neutralizing antibody levels of 1:2 have been shown to be protective.^45,46^

Since a dichotomous seroresponse may be the critical determinant of protection by the rVSV-Nipah vaccine (as for rVSV-EBOV^8,43^) It would be advantageous to adopt a method for reliable determination of neutralizing antibodies at very low concentration and low affinity. In addition to minimizing the starting dilution of serum, it may be useful to compensate for heat-inactivation, a routine process that destroys complement and other heat-labile co-factors that can contribute to neutralization.^47^ In addition to adding back complement or fresh serum factor (which are not identical^71^), other methods of enhancing the sensitivity of neutralization could be explored, in particular addition of anti-IgG or IgM, which has been useful for detection of neutralization of mumps virus, another paramyxovirus.^48,49,50^ Additionally a 50% cut-off for the VNA rather than 80% could increase sensitivity.

Retesting of AGM samples from the original study of immune correlates of protection^25^ using the VNA, which is now the method being applied in clinical trials of PHV02, enables bridging of the nonclinical and clinical data. The re-analysis confirmed that a detectable neutralization titer signaled protective immunity. A more conservative threshold -- seroconversion (a ≥ fourfold rise from baseline, i.e. from LLOQ/2 = 6 to LLOQ/2 × 4 = 24) is suggested. Regression analysis with noise indicated that the probability of survival at a VNA of 24 before exposure to NiV is 74.8% (95% CI 37.7%, 93.6%) (Fig. 6). In clinical development of PHV02 vaccine, a prospective efficacy target is used of ≥ 70% for the lower bound of the 90% CI of the seroconversion rate, which corresponds quite closely to the probability of survival in the AGM model.

Given the expected use of the vaccine for rapid outbreak control and post-exposure prophylaxis (PEP), it will be important to investigate more intensively the 7–14 day interval following vaccination during which antibodies reach high levels. The three-day interval 8–10 days after vaccination appears to be critical time for appearance of virus-specific antibodies. This is consistent with other live viral vaccines for which time to appearance of neutralizing antibodies (as the key metric of protection) has been evaluated including rVSV-EBOV^95^ and yellow fever^51^, as well as antibodies following natural infection with SARS-CoV-2.^52^ The time to appearance of neutralizing antibodies has special importance for quarantinable infections like yellow fever and Covid-19 as the basis for “immunological passports” and vaccination certificates for travel that ultimately will apply to Nipah vaccine(s). Information on the kinetics of the protective immune response is also important for planning outbreak intervention.

The protection seen in Study 2 animals vaccinated on Day – 7 which lacked a substantial neutralizing response at the time of challenge suggests that protection may have a polyfunctional basis. In a previous study of the rVSV vaccine expressing NiV G (Malaysia), Prescott et al. reported increased CD8 + T cells expressing granzyme B or interferon-g in vaccinated vs. control animals at 3- and 7-days post-challenge, respectively.^53^

It is likely that a time-to-protection study, if repeated and using optimized neutralization methods (low starting dilution, and possibly enhancement with complement, fresh serum factor or anti-IgG), would reveal consistent pre-challenge low level antibodies in animals vaccinated shortly before challenge, if not at −7 days, then at- 8 to −10 days. This could be a productive avenue of research to confirm that low antibody titers and a serological ‘take’ are indicative of protection. Confirmation that seroresponse (a > 2-fold rise) is protective would support the suggested more conservative seroconversion (> 4-fold) rise from LLOQ/2 (6) to a VNA titer of 24 that minimizes the influence of lab test variation. This conclusion is consistent with the observations from clinical trials of hepatitis B, rabies, Japanese encephalitis, yellow fever, and Ebola vaccines indicating that an increased from negative to above the LLOQ of the neutralization method is a surrogate of protection.

A notable finding in our studies was the appearance of clinical signs of illness and lung Xray changes in animals that were vaccinated and had substantial neutralizing antibody titers. These animals survived and the clinical signs were milder than in control animals and resolved within 7–10 days (Fig. 4). Breakthrough clinical infections in vaccinated persons are described for a wide variety of vaccines, including highly effective vaccines like measles^54^, mumps^55^, varicella^56^, and hepatitis vaccines^57,58^, generally with milder signs than in unvaccinated persons as seen in the case of PHV02 vaccinated monkeys following Nipah virus challenge. In our study, the frequency of breakthrough clinical signs decreased as the interval between vaccination and challenge was lengthened and antibody titers increased.

Finally, although encephalitis is a prominent clinical manifestation of NiV disease in humans^59^, the disease in AGMs is predominantly acute respiratory distress, probably because death/sacrifice precedes significant brain inflammation. Additionally, challenge in the AGM model involved direct pulmonary (intratracheal) inoculation of NiV. Despite the lack of prominent clinical encephalitis, viral RNA and virus were observed in brains of control AGMs at necropsy.^33^ Others have shown that NiV may persist in brains of surviving AGMs^60^, and relapsing encephalitis has been reported in human survivors.^61^ if the AGM model were refined to allow evolution of encephalitic manifestations, it will be necessary to revisit the immune correlate of protection. Since immunoglobulins do not cross the (intact) blood brain barrier.^62^ Even a low level of neutralizing antibody may be effective in preventing virus transcytosis to the brain from the bloodstream. However, NiV neuroinvasion may occur via direct cranial nerve axonal transfer or via the olfactory apparatus,^63, 64^ enabled by the IN route of inoculation under anesthesia of a large volume that could reach the olfactory neuroepithelium located high up in the nasal cavity.

## 5. Conclusions

Neutralizing antibody response to a rVSV-Nipah live, attenuated vaccine (PHV02) was shown to correlate with survival in a 100% lethal Nipah virus challenge model in African green monkeys. Neutralizing antibodies were measured by a pseudovirus luminescence reduction (VNA) method. A low level of antibody was sufficient to protect against death. Seroconversion (≥ fourfold rise in titer from negative imputed as LLOQ/2) is proposed as a conservative putative surrogate of efficacy. These data in nonhuman primates can be useful in bridging results to antibody responses to PHV02 vaccine in humans.

## Figures and Tables

**Figure 1:**
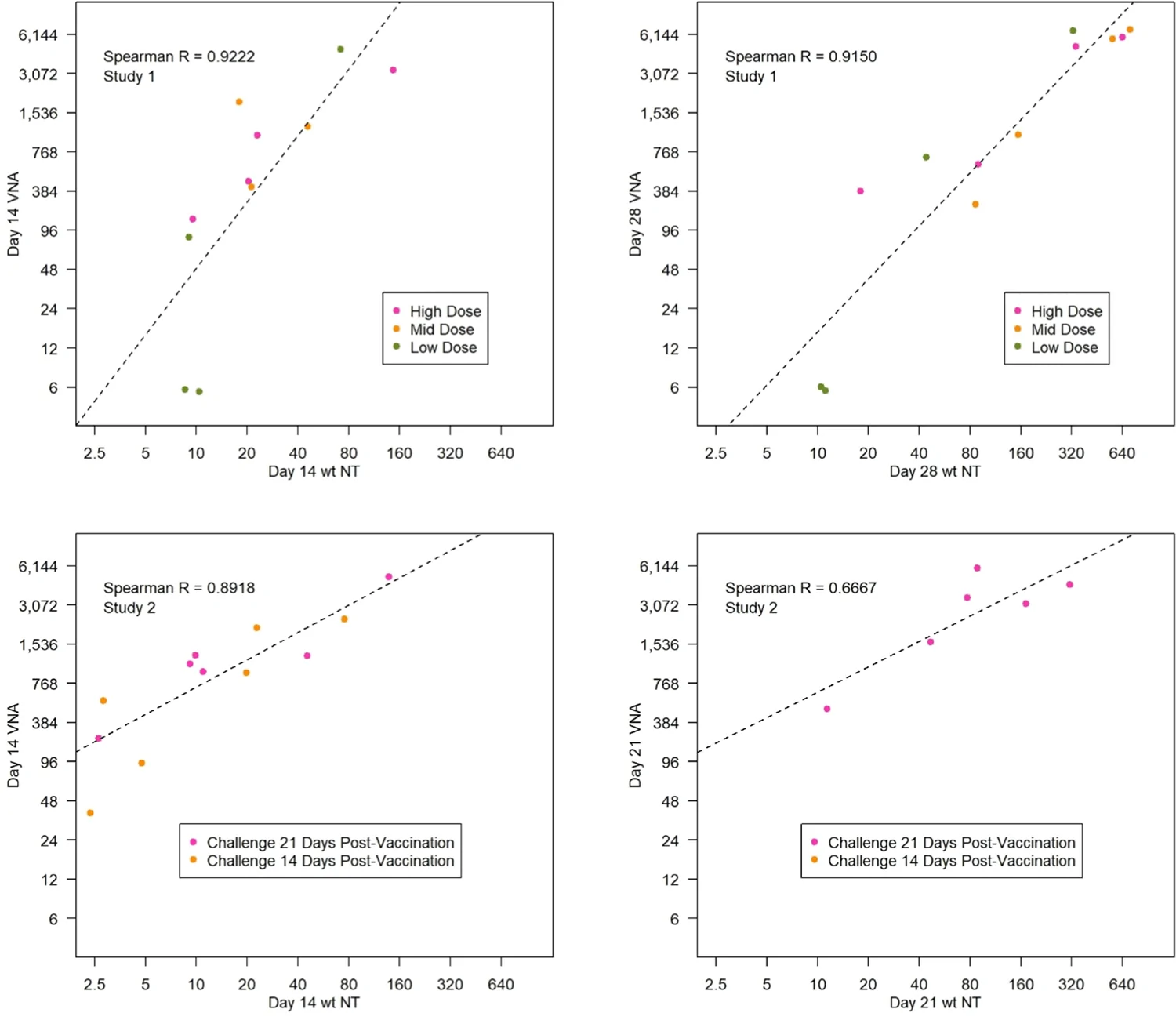
Linear regression analysis of wt NT vs. VNA titer of AGM sera in Study 1 (top) and Study 2 (bottom) by day after vaccination

**Figure 2:**
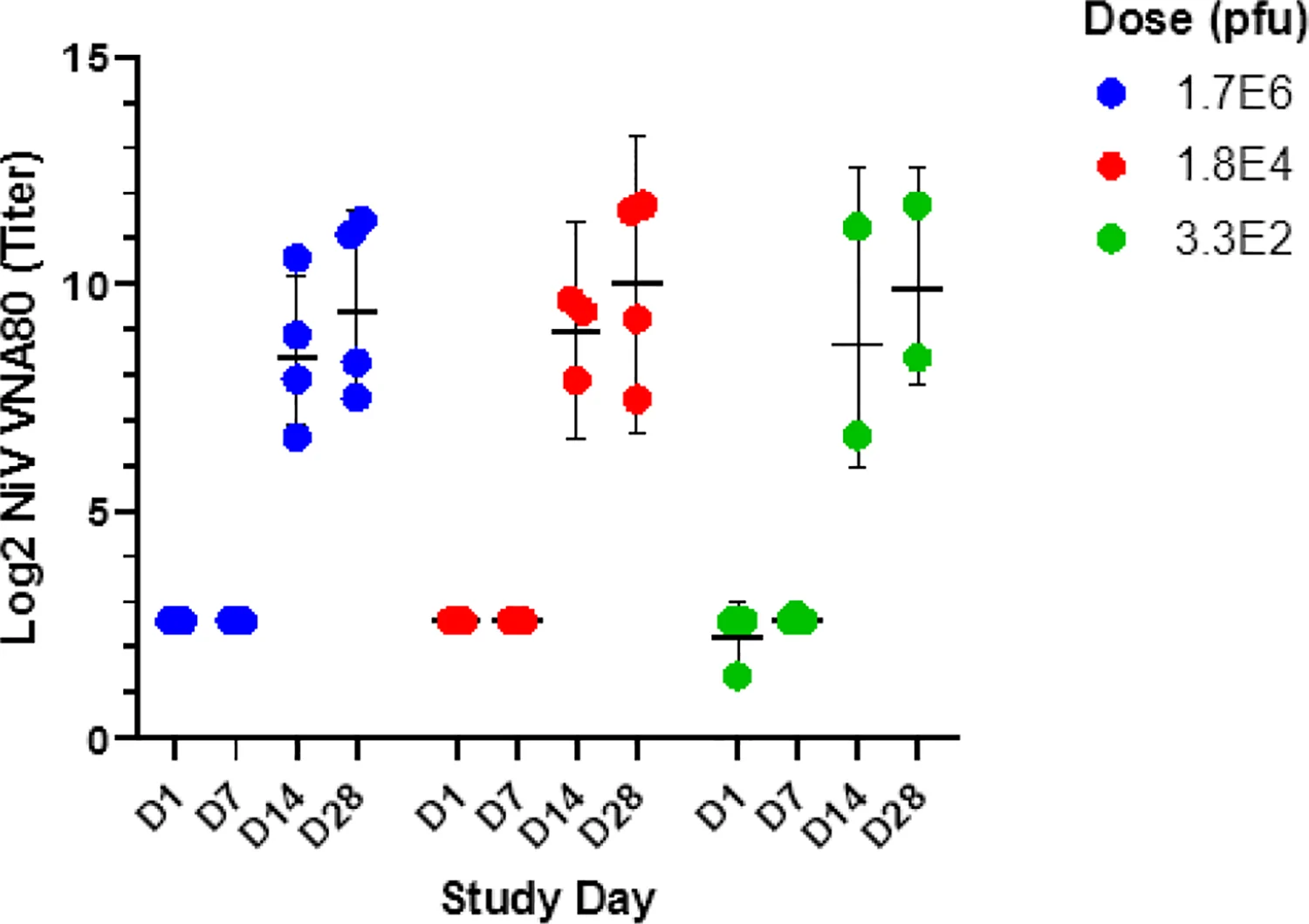
VNA antibody titers in individual survivor AGMs (GMT and 95% CI) by dose group and day after vaccination in Study 1. For statistical evaluation, VNA titers are shown as log2 values. Excluding from analysis the two non-responders in the Low dose group in Study 1, there were no differences in GMT between the three dose groups (nested one-way ANOVA for dose effect p=0.9952)

**Figure 3:**
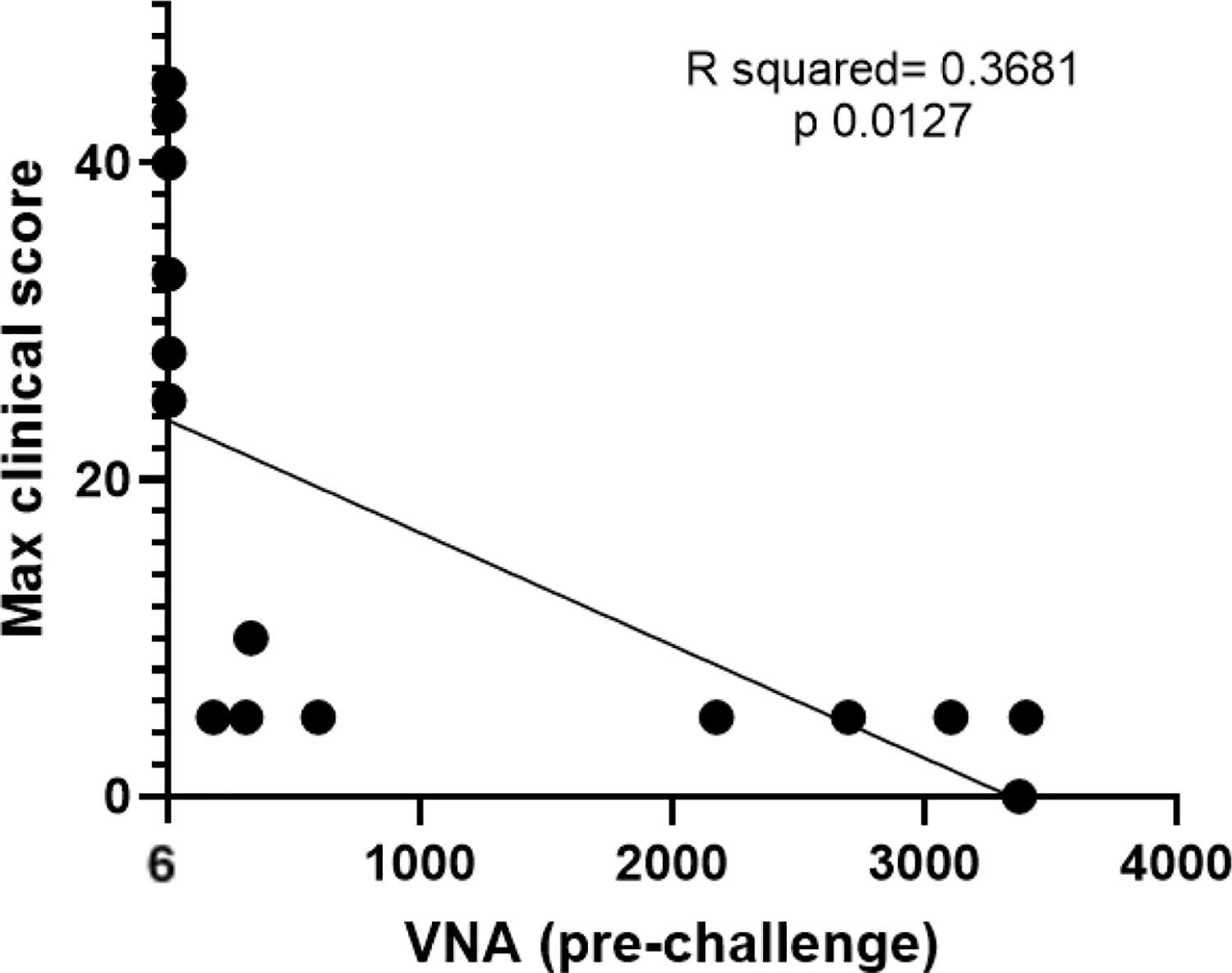
Correlation between VNA titer before challenge and maximum clinical score, all AGMs in Studies 1 and 2.

**Figure 4:**
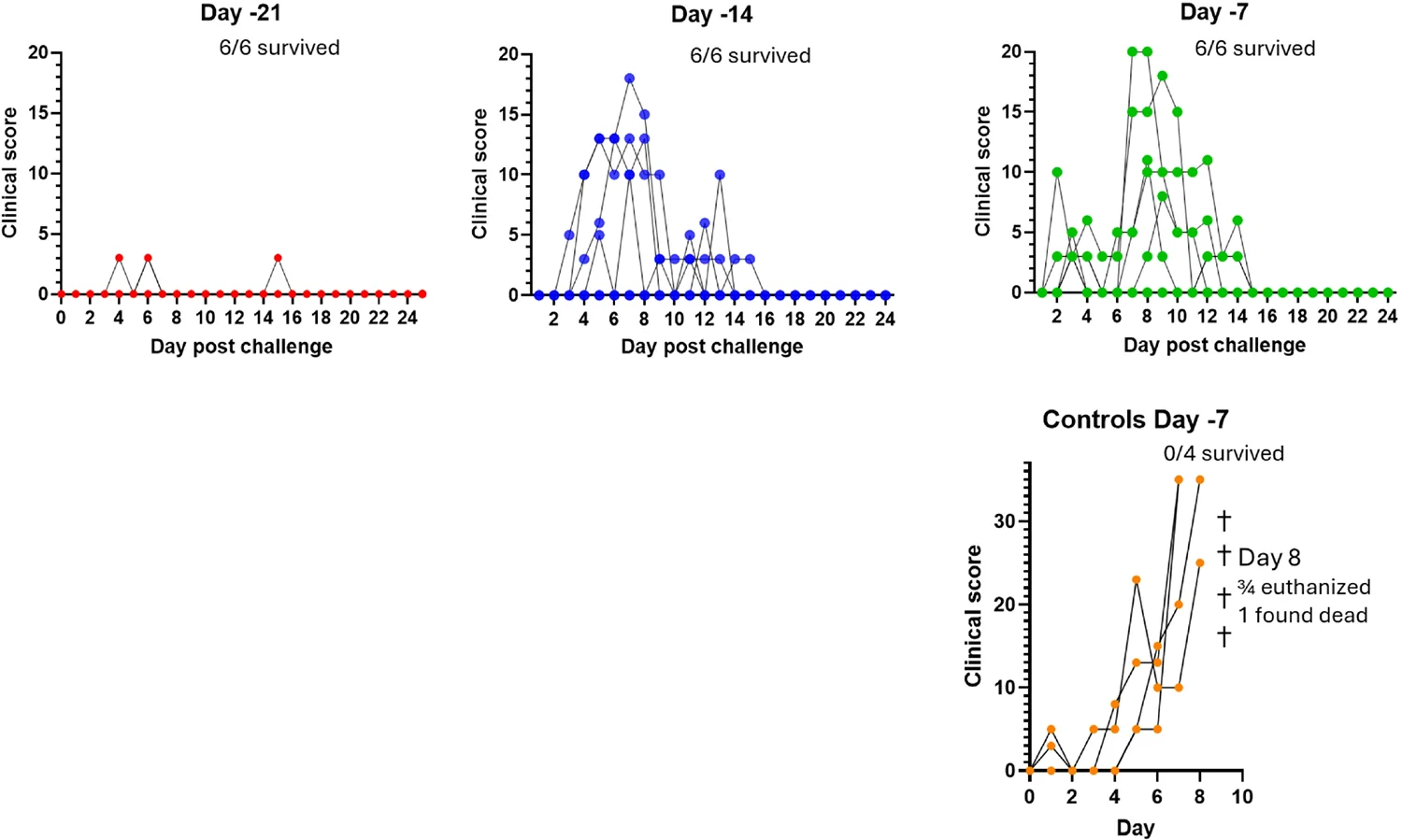
Clinical scores, individual AGMs by interval between vaccination and NiV challenge, Study 2. All animals vaccinated with PHV02 survived, but when the interval between vaccination and challenge was short, clinical illness occurred lasting 14–15 days, followed by recovery.

**Figure 5:**
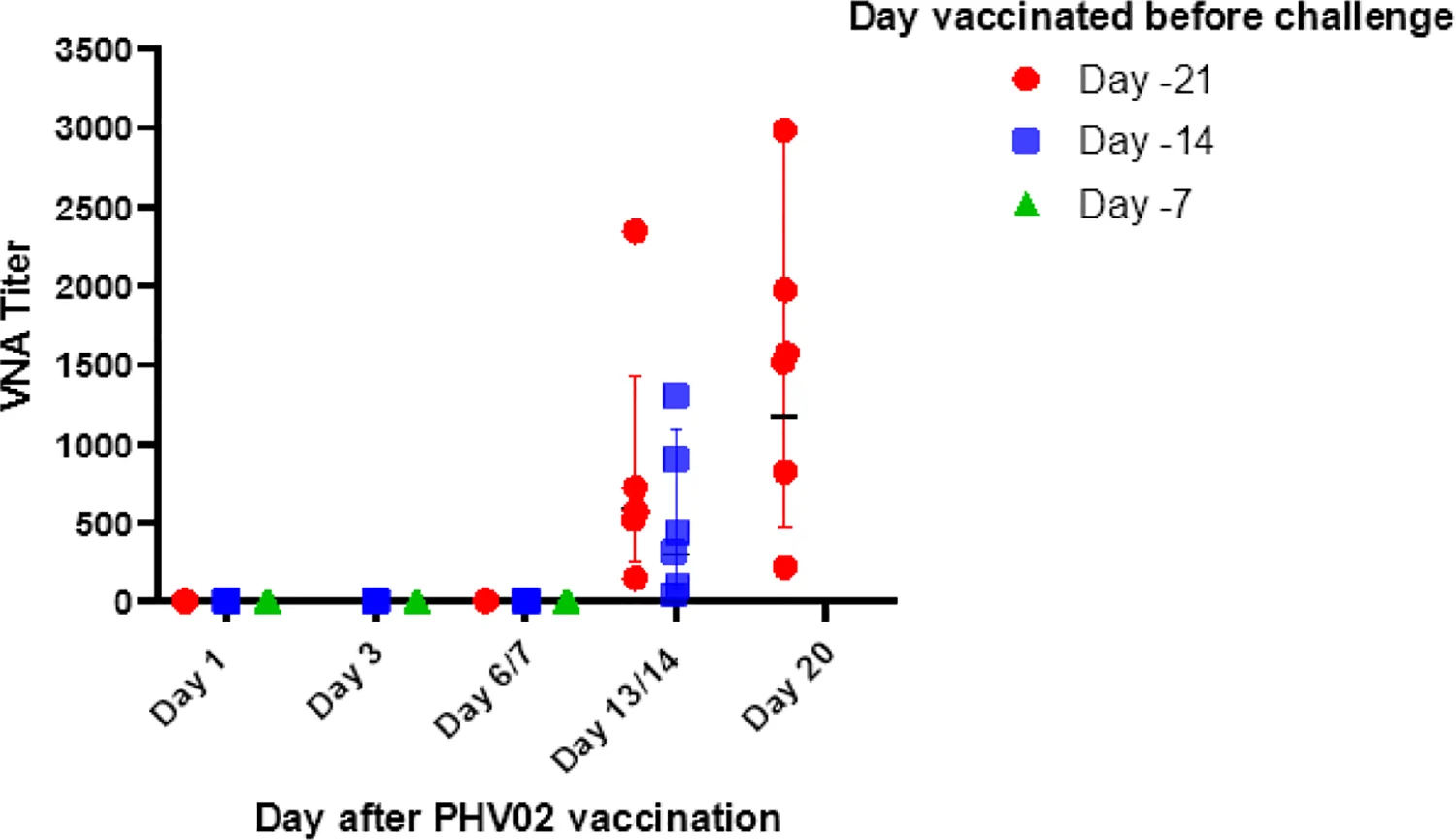
VNA titers, individual AGMs grouped by day of vaccination with respect to NiV challenge and test day after vaccination, GMT ± GSD

**Figure 6:**
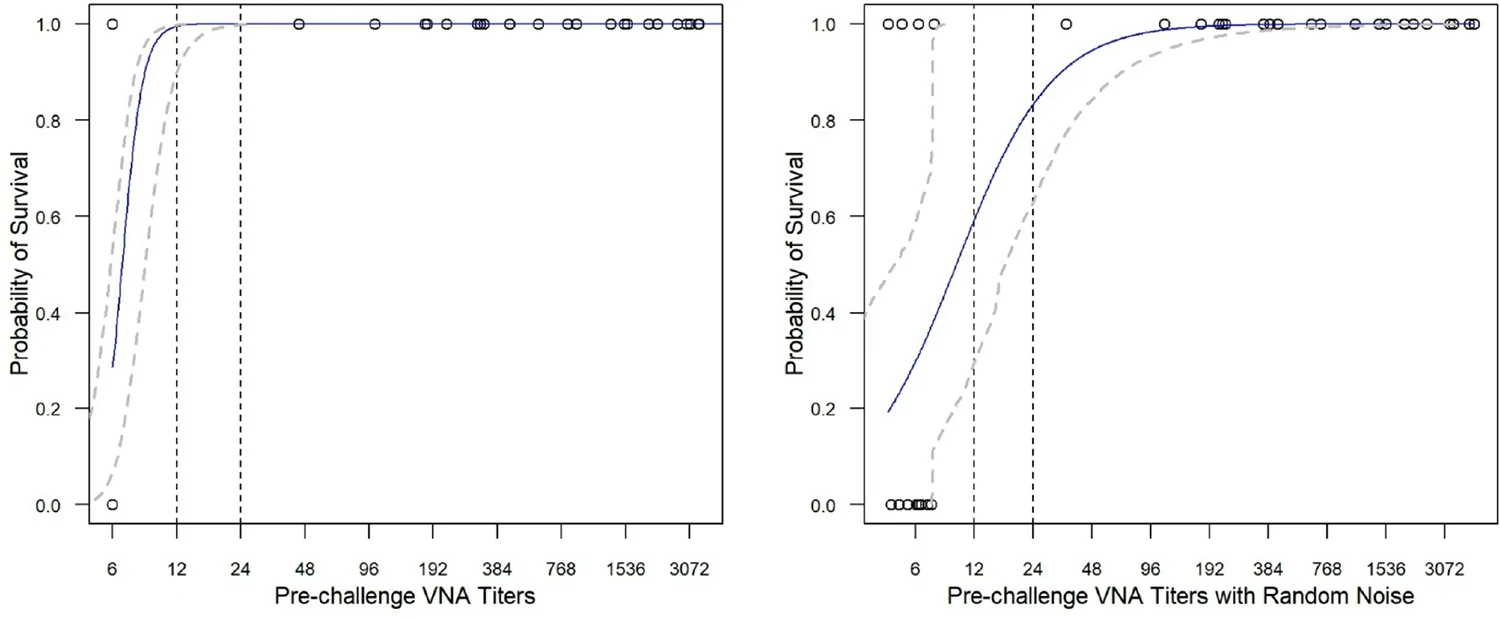
Predicted survival curves and their 95% confidence interval for a given pre-challenge log2-transformed VNA titer for the pooled data consisting of all 38 monkeys, Studies 1 and 2, without noise (left) and with random noise added to the log-2 transformed antibody data (right).

**Table 1:** Study 1 AGMs vaccinated with PHV02 or rVSV-EBOV (control) 28 days before IT/NT challenge with 2 × 10^5^ TCID50 of NiV (Bangladesh): neutralizing antibody titers by VNA by day with respect to challenge, outcome, and post-challenge viremia.

Monkey	Vaccine and dose	Neutralizing antibody titer VNA 1/dil				Outcome (time of sacrifice)	Nipah viremia Day + 1, +3, +7	Peak challenge viremia (log10 copies/mL) [Day]	Maximum clinical score^2^	Maximum lung radiograph score^3^	≥ 4-fold wt NT antibody rise post challenge^4^
	Day with respect to challenge	Day − 28	Day − 21	Day − 14	Day − 1						
	Day with respect to vaccination	Day 0	Day 7	Day 14	Day 28						
NiV 123	PHV02 High dose (1.7 × 10^6^ pfu)	< 12	< 12	**1509**	**2696**	Survived	No	-	5	2	No
NiV 124		< 12	< 12	**99**	**180**	Survived	No	-	5	2	Yes
NiV 125	+	< 12	< 12	**239**	**310**	Survived	No	-	5	2	Yes
NiV 126		< 12	< 12	**466**	**2175**	Survived	No	-	5	2	No
	**GMT (GSD)** ^5^	6 (1)	6 (1)	**359.2 (3.15)**	**756.3 (3.92)**						
	**GMFI (GSD) from baseline** ^5^	-	**1 (0)**	**59.9 (5.89)**	**126.1 (7.68)**						
	**GMFI (GSD) from D-14**			-	**2.1 (1.74)**						
NiV 119	PHV02 Mid dose (1.8 × 10^4^ pfu)	< 12	< 12	**803**	**3402**	Survived	No	-	5	2	No
NiV 120		< 12	< 12	**667**	**3104**	Survived	No	-	5	2	Yes
NiV 121		< 12	NT	NT	**176**	Survived	No	-	5	2	No
NiV 122		< 12	< 12	**235**	**597**	Survived	No	-	3	1	No
	**GMT (GSD)** ^5^	6 (1)	6 (1)	**501.2 (6.22)**	**1026.3 (6.93)**						
	**GMFI (GSD) from baseline** ^5^		-	**83.5 (1.93)**	**171.1 (4.14)**						
	**GMFI (GSD) from D-14**			-	**3.7 (1.39)**						
NiV 115	PHV02 Low dose (< 6.6 × 10^2^ pfu)	< 12	< 12	< 12	< 12	Died (Day 7)	Yes	6.0 [+ 7]	33	13	-
NiV 116		< 12	< 12	< 12	< 12	Died (Day 7)	Yes	4.3 [+ 7]	25	13	-
NiV 117		< 12	< 12	**101**	**331**	Survived	No	-	10	1	No
NiV 118		< 12	< 12	**2458**	**3374**	Survived	No	-	0	3	No
	**GMT (GSD) (pos. only)** ^5^	6 (1)	6 (1)	**498.3 (6.21)**	**1056.8 (6.96)**						
	**GMFI (GSD) from baseline** ^5^		-	**83.0 (5.16)**	**171.1 (4.14)**						
	**GMFI (GSD) from D-14** ^5^			-	**2.1 (1.85)**						
NiV 111	rVSV-EBOV Control (2 × 10^7^ pfu)	< 12	< 12	< 12	< 12	Died (Day 7)	No	-	43	8	-
NiV 112		< 12	< 12	< 12	< 12	Died (Day 7)	Yes	5.7 [+ 7]	40	7	-
NiV 113		< 12	< 12	< 12	< 12	Died (Day 7)	Yes	6.3 [+ 7]	28	8	-
NiV 114		< 12	< 12	< 12	< 12	Died (Day 7)	Yes	6.8 [+ 7]	45	12	-
	**GMT**	6 (1)	6 (1)	6 (1)	6 (1)						
	**GMFI (from baseline)** ^5^		-	-	-						
	**GMFI (from D-14)**				-						

Footnotes:

1 AGMs were also tested by VNA on Days – 27 and – 25, and all sera had titers < 12

2,3 see Materials and Methods for description of scoring system.

4 Post-challenge NiV neutralizing antibody levels measured at Rocky Mountain Laboratories by CPE-based assay using wild-type NV and reported previously in reference^31^.

5 For analysis of GMT and GMFI, the LLOQ is divided by 2, i.e. a titer of < 12 is given a value of 6

**Table 2:** Study 2 (time-to-protection): AGMs vaccinated IM with 2 × 10^7^ pfu PHV02 21, 14, or 7 days before IN/IT challenge with 2 × 10^5^ TCID50 NiV (Bangladesh). Con received an equivalent dose of rVSV-EBOV 7 days before challenge. VNA neutralizing antibody titers by day with respect to challenge, GMT, GMFI outcome, post-challenge viremia and antibody response are presented.

AGM	Vaccine (dose) Day vaccinated before challenge	VNA schedule	VNA 1/dil				Outcome (Day of death/sacrifice)	NiV Viremia	Peak challenge viremia (copies/mL) [Day]	Maximum clinical score	Maximum lung radiograph score	≥ 4-fold wt NiV antibody rise post challenge
		Day after vaccination	Day 1	Day 7	Day 14	Day 20						
NiV 127	PHV02 (2 × 10^7^ pfu) Day − 21		< 12	< 12	**150**	**221**	Survived	No	-	0	5	Yes
NiV 128			< 12	< 12	**576**	**1570**	Survived	No	-	0	4	No
NiV 129			< 12	< 12	**575**	**2983**	Survived	No	-	3	1	No
NiV 130			< 12	**10**	**524**	**823**	Survived	No	-	0	3	Yes
NiV 131			< 12	< 12	**2344**	**1973**	Survived	No	-	3	1	No
NiV 132			< 12	< 12	**710**	**1517**	Survived	No	-	0	1	No
		GMT (GSD)^2^	**6 (1)**	**6.53 (1.23)**	**592.6 (2.40)**	**1168.8 (2.50)**						
		GMFI (GSD) from baseline^2^	-	**1.09 (1.23)**	**90.7 (2.49)**	**194.8 (2.50)**						
		GMFI (GSD) from Day 14^2^	-	-	-	**2.32 (1.85)**						
		Day after vaccination	**Day 1**	**Day 3**	**Day 7**	**Day 13**						
NiV 133	PHV02 (2 × 10^7^ pfu) Day −14		< 12	< 12	**8**	**1306**	Survived	No	-	0	2	No
NiV 134			< 12	< 12	< 12	**319**	Survived	No	-	0	1	Yes
NiV 135			< 12	< 12	< 12	**102**	Survived	No	-	18	1	Yes
NiV 136			< 12	< 12	< 12	**45**	Survived	No	-	13	2	Yes
NiV 137			< 12	< 12	< 12	**440**	Survived	No	-	5	4	Yes
NiV 138			< 12	< 12	< 12	**901**	Survived	No	-	13	2	Yes
		GMT (GSD)^2^	**6 (1)**	**6 (1)**	**6.29 (1.12)**	**302.0 (3.62)**						
		GMFI (GSD) from baseline)^2^	-	**1 (1)**	**1.05 (1.12)**	**50.3 (3.62)**						
		Day after vaccination	Day 0	Day 1	Day 3	Day 6						
NiV 141	PHV02 (2 × 10^7^ pfu) Day − 7		< 12	< 12	< 12	QNS	Survived	No	-	10	2	Yes
NiV 142			QNS	< 12	< 12	**QNS** ^1^	Survived	No	-	3	2	Yes
NiV 143			< 12	< 12	< 12	< 12	Survived	No	-	11	3	Yes
NiV 144			< 12	< 12	< 12	< 12	Survived	Yes	2.91 [D + 7]	8	2	Yes
NiV 145			< 12	< 12	< 12	< 12^1^	Survived	No	-	18	2	Yes
NiV 146			< 12	< 12	< 12	< 12	Survived	No	-	20	1	Yes
		GMT (GSD)	6 (1)	6 (1)	6 (1)	6 (1)						
		GMFI (GSD) (from baseline)^2^	-	1 (1)	1 (1)	1 (1)						
		Day after vaccination	Day 0	Day 1	Day 3	Day 6						
NiV 139	rVSV-EBOV Control (2 × 10^7^ pfu) Day – 7		< 12	< 12	< 12	< 12	Died (Day 8)	No	5.9 [D + 7]	25	18	-
NiV 140			< 12	< 12	< 12	< 12	Died (Day 8)	Yes	5.5 [D + 7]	35	3	-
NiV 147			< 12	< 12	< 12	< 12	Died (Day 8)	Yes	5.6 [D + 7]	35	17	-
NiV 148			< 12	< 12	< 12	< 12	Died (Day 8)	Yes	5.5 [D + 7]	35	2	-
		GMT (GSD)	6 (1)	6 (1)	6 (1)	6 (1)						
		GMFI (GSD) (from baseline)	-	1 (1)	1 (1)	1 (1)						

1 These AGMs had a wild-type NiV neutralization titer of 10 on Day 6 (1 day before challenge)^31^

2 For analysis of GMT and GMFI, the LLOQ is divided by 2, i.e. a titer of < 12 is given a value of 6

## Data Availability

All data supporting the findings of this study are available within the paper or on request to the corresponding author
